# NFP: An R Package for Characterizing and Comparing of Annotated Biological Networks

**DOI:** 10.1155/2017/7457131

**Published:** 2017-02-09

**Authors:** Yang Cao, Wenjian Xu, Chao Niu, Xiaochen Bo, Fei Li

**Affiliations:** ^1^Department of Biotechnology, Beijing Institute of Radical Medicine, 27 Taiping Road, Haidian District, Beijing 100850, China; ^2^Tianjin Institute of Health & Environmental Medicine, 1 Dali Road, Heping District, Tianjin 300050, China

## Abstract

Large amounts of various biological networks exist for representing different types of interaction data, such as genetic, metabolic, gene regulatory, and protein-protein relationships. Recent approaches on biological network study are based on different mathematical concepts. It is necessary to construct a uniform framework to judge the functionality of biological networks. We recently introduced a knowledge-based computational framework that reliably characterized biological networks in system level. The method worked by making systematic comparisons to a set of well-studied “basic networks,” measuring both the functional and topological similarities. A biological network could be characterized as a spectrum-like vector consisting of similarities to basic networks. Here, to facilitate the application, development, and adoption of this framework, we present an R package called NFP. This package extends our previous pipeline, offering a powerful set of functions for Network Fingerprint analysis. The software shows great potential in biological network study. The open source NFP R package is freely available under the GNU General Public License v2.0 at CRAN along with the vignette.

## 1. Introduction

The advances in high-throughput experimental technology and system biology have led to an explosive growth of biological interaction data in both size and complexity, and promoting network-based methods become the key to understand biological activities [1, 2]. Various biological networks (such as gene regulatory, metabolic, and protein-protein networks) that represent interactions in molecular biology exist for many species [3, 4]. On the other hand, many online repositories like Kyoto Encyclopedia of Genes and Genomes (KEGG) [5], Reactome [6], and Human Protein Reference Database (HPRD) [7] were created for hosting large amount of well-studied biological networks. These networks provide a good basis for knowledge-based exploration of biological networks with plain understanding. However, recent research on molecular network requires a higher mathematical level and cannot provide a uniform understanding. In addition, current algorithms on systematic network analysis are mainly based on the topological structure and neglect the functional interactions of molecular in the network.

To address this, we recently described a framework to decipher biomedical networks by making systematic comparisons to reference sets of well-studied “basic networks” [8]. Our method measures both functional and structural similarities between a query network and each basic network and provides a novel representation of network differentiation known as biological spectra, which is termed as Network Fingerprint. Given a set of basic networks *P* = (*P*_1_, *P*_2_,…, *P*_*n*_), a query network *G* can be characterized as a Network Fingerprint *S* = (*s*_1_, *s*_2_,…, *s*_*n*_), where *s*_*i*_ = sim(*G*, *P*_*i*_) represents the similarity score between *G* and *P*_*i*_. It would help us to compare multiple molecular networks in a systematic way, and it is very favorable for deciphering the consistency and heterogeneity of biological systems [9]. We showed that this approach can be used to describe the relationship between multiple disease networks and its related pathways and to visually compare and parse different diseases by generating a fingerprint overlay. Here, to facilitate the application and development of this algorithm, we present a freely available, easy-to-use R package called NFP that implements Network Fingerprint framework as a small number of easy-to-use functions. This package allows generation of basic networks from the current biological network databases, computation of biological Network Fingerprint referring to basic networks, and visualization of Network Fingerprint. See the vignette of NFP for full functions and their applications. An installation guide and additional generic use cases for NFP are described in the package vignette and the website https://yiluheihei.github.io/NFP/.

## 2. Methods and Implementations

**Figure 1:**
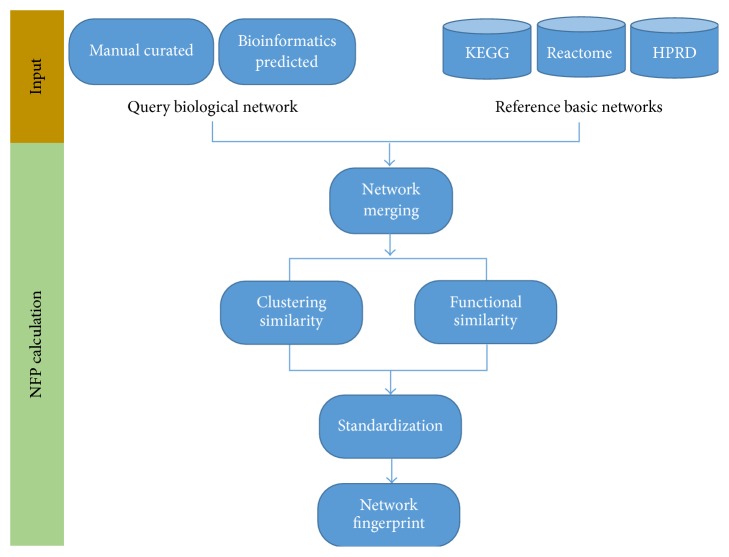
A typical analysis pipeline supported by the NFP package.

The NFP R package compares a biological network to preset basic networks and characterizes this network as biological Network Fingerprint. Our package utilizes an algorithm, from a network merging scheme, to measure both functional and structural similarities between the query network and each preset basic network based on the Gene Ontology (GO) and affinity propagation (AP) clustering algorithm [10]. The similarities are then standardized by a randomization procedure, forming a characterized Network Fingerprint of the query network. The package employs igraph [11] and graph [12] to store the biological networks for making network analysis efficient, portable, and easy to use. Furthermore data manipulation and visualization functions are built upon the tidyverse packages including dplyr [13], ggplot2 [14], stringr [15], tidyr [16], and plyr [17] to make our package more efficient and easy to extend. In addition, two versatile R S4 classes,* NFPRefnet-class* and* NFP-class*, are defined to store and manipulate the complex reference basic networks and scored Network Fingerprint. All R source code is freely available on GitHub (see “Data Access” section). In the following we will explain the two main features of the package: the basic networks generation and Network Fingerprint calculation.  Figure 1 shows the analysis pipeline of NFP.

### 2.1. Basic Networks Generation

Basic networks generation is the first and basic step of NFP. As the importance of coordinate system to analytic geometry, the choice of basic networks in NFP is vital for anchoring the shape of the Network Fingerprint. Kyoto Encyclopedia of Genes and Genomes (KEGG) pathway mappings are the most common used and well-characterized biological networks, which represent basic knowledge on molecular interaction and reaction networks for various processes [5]. These basic networks could be obtained with the KEGG REST API. Moreover, basic networks may be classified into several categories; for example, KEGG pathway networks are divided into seven categories such as metabolism, genetic information processing, and cellular processes. NFP provides function* load_kegg_refnet* to import KEGG biological networks, which could be set as the reference basic networks in the following NFP calculation. Then we can directly decipher the disease pathways by means of constructing the disease Network Fingerprint based on KEGG pathways. As mentioned above, basic networks data is saved as an* NFPRefnet* object. And five methods for this class, including* net*,* group*,* subnet*,* refnet_name*, and* show*, are created for allowing users to easily customize their basic networks. Specially, this S4 class also allows users to create personalized basic reference networks. For more details see the help manual of* NFPRefnet-class*.

### 2.2. Network Fingerprint Calculation

Network Fingerprint calculation, measuring interested biological network based on a set of given reference network systems (basic networks), is the core feature of NFP. The similarity between a query network and each basic network is calculated as the following steps: network merging, similarity scoring, and standardization.

First, we merge the two compared networks *G*_1_ = {*V*_1_, *E*_1_} and *G*_2_ = {*V*_2_, *E*_2_} into one fully connected network according to *G*_*m*_ = (*V*_1_ ∪ *V*_2_, *E*_1_ ∪ *E*_2_). Network merging method proceeds in two steps: (a) all the nodes and edges from two compared networks are pooled to form a merged network; (b) common nodes (defined as items having the same names) are replaced into a single node that inherited all the interactions from the common nodes and common edges (defined as items connecting the same two nodes) are merged into a single edge. Second, GO-based functional semantic similarity has been widely used for function prediction/validation and protein-protein interaction prediction/validation. Several approaches are available to measure the semantic similarity. The most common measures are Resnik's [18], Lin's [19], and Jiang and Conrath's [20], which are node-based measures based on information content (IC). Many studies have been performed to test and evaluate the three measures and conducted the pairwise measures using Resnik's term similarity in biological process that outperformed Lin's and Jiang and Conrath's methods in all studies except family similarity [21]. Thus here Resnik's similarity is taken for weighing the merged network to measure the functional similarity, which is defined as *S*_*m*_ and the weighted adjacency matrix *S*_*m*_ of *G*_*m*_ is calculated as follows:

(1) Smi,j=1V1Vm+V2Vm∑i∈V1Vm,j∈V2Vmfmax⁡Si,j+∑j∈V2Vm,i∈V1Vmfmax⁡Si,j,

where *V*^1^*V*^*m*^ represents the number of nodes in both *G*_1_ and the merged network *G*_*m*_ and *V*^2^*V*^*m*^ represents similarity.

Subsequently, AP clustering algorithm is used for grouping the nodes to measure the structural similarity. The local similarity score of each cluster is defined as follows:

(2) LSk=1nVk1+nVk2∑i∈Vk1,j∈Vk2max⁡Smi,j+∑j∈Vk2,i∈Vk1max⁡Smi,j,

where LS_*k*_ represents the local similarity score, *V*_*k*_^1^ represents the nodes in both *G*_1_ and the nodes in cluster *k* but not in *G*_2_, and *n*(*V*_*k*_^1^) represents its number, same as in *G*_2_. The global similarity score between two networks is defined as the mean similarity over all clusters. Finally, the similarity score calculated above may be biased since it may partly depend on network topological properties. To eliminate the underlying differences of topological weights, randomized networks were generated to standardized the similarity score according to Maslow's method [22].

Our package provides* cal_sim_score*, the core as well as the most important function, for Network Fingerprint calculation. It takes three arguments, a* graphNEL* object to represent the query network, an* NFPRefnet* object to store basic networks, and an integer to indicate the number of randomized networks for standardization. It returns an* NFP-class *object containing the unstandardized, randomized, and standardized Network Fingerprint based on basic networks and clusters information. NFP provides five methods to easily manipulate the* NFP* object, giving a better understanding of Network Fingerprint. For example, function* plot_NFP* shows an overview of the Network Fingerprint.

Please note that standardization is the most time-consuming process in Network Fingerprint calculation. To improve the efficiency of NFP without affecting the results of standardized, the default standardization parameter is set to 100. Users can also adjust randomization time of background network based on requirements for the precision of Network Fingerprint.

## 3. Results

We have previously successfully utilized Network Fingerprint framework for studying disease networks and have shown that it provides tools for better understanding of biomedical networks based on the KEGG signaling networks [8]. This study demonstrates the applicability of NFP to disease networks comparison, classification, and relationships with signal pathways. To illustrate the use of NFP, below we show an example on FOXM1 pathway in breast cancer that consists of transcriptional cancer drivers and risk genes [23].

Load the NFP package, KEGG signal pathway networks as basic networks and the FOXM1 pathway network data as follows:

## Install the NFP package
source("http://bioconductor.org/biocLite.R")
biocLite ("NFP")
## load the library and install the data package
library (NFP)
install_data_package ()
## load the sample FOXM1 network from the github
load (url( *＇* https://yiluheihei.github.io/NFP/paper_results/sample_FOXM1_Kwoneel.rdata *＇* ))
FOXM1_net <- igraph::igraph.to.graphNEL (sample_FOXM1_Kwoneel)
## As mention above, NFP calculation may take several hours due to the large nperm para
FOXM1_nfp <- calc_sim_score (FOXM1_net, kegg_refnet, nperm = 1000)

The above command calculates the spectrum-like Network Fingerprint of FOXM1 pathway, constructed with the similarity scores between this pathway and each KEGG signal pathway. The FOXM1 transcription factor is a key regulator of various biological processes, such as DNA replication and repair and cell cycle progression, and plays an important causal role in the development of aggressive breast cancer [24, 25]. Consistently (for more details see Table 1), FOXM1 pathway has higher similarity scores with DNA replication and repair pathways in our KEGG signal basic networks, including DNA replication (2.62), Base excision repair (3.02), Nucleotide excision repair (2.42), Homologous recombination (5.74), Nonhomologous end-joining (1.88), and Fanconi anemia pathway (2.82). Moreover, the similarity score to cell cycle pathway is also very high (2.40). All of them are in the top 10 percent of FOXM1's Network Fingerprint. It should be noted that the results will be slightly different each time the Network Fingerprint is calculated, since randomized networks were used to standardize the Network Fingerprint to eliminate the differences of topological weights. The sample data and source code for this sample can be downloaded from https://yiluheihei.github.io/NFP/.

Additionally, NFP included a function* plot_NFP* to visualize a single Network Fingerprint. It gives an overview of Network Fingerprint along all basic networks, and different groups of basic networks are displayed in different colors. Furthermore, we created another function* plot_NFPlist *in NFP to show the comparison and differentiation among different Network Fingerprints: plot_NFP (FOXM1_nfp).

## 4. Conclusions

The NFP package allows easily performing Network Fingerprint analysis of biological networks, providing a systematic understanding of the ever-increasing biological networks based on the well-characterized networks. In addition, the package includes tools that offer understandable visualization of Network Fingerprint and the functions for manipulation NFP data. We have shown that NFP gives a system understanding of FOXM1 transcriptional driver pathway, highlighting the potential of our package. The NFP package, along with previously introduced framework, completes the suite of Network Fingerprint analysis tools accessible to researchers with any level of computer and mathematical expertise. Unfortunately, the current version of NFP has two primary limitations that we will be addressed in the future. First, metabolic networks have not been fully understood. For example, there are ambiguity and promiscuity in enzyme reaction, suggesting the existence of many hidden metabolic reactions that are important for metabolic pathways study [26]. The metabolic network models we used usually contain gaps that prevent the production of one or more components of the reaction. Secondly, our package could be time-consuming because of slow computation of the similarity score standardization, especially for large number of networks. We plan to optimize the metabolic network models and our package for exact and efficient analysis.

## Figures and Tables

**Table 1:** The top ten percent Network Fingerprint and the corresponding reference KEGG pathways.

Reference networks	Network Fingerprint
Homologous recombination	5.738022
Base excision repair	3.024813
Fanconi anemia pathway	2.817877
SNARE interactions in vesicular transport	2.678513
DNA replication	2.625542
Nucleotide excision repair	2.416703
Basal transcription factors	2.399798
Cell cycle	2.392328
Proteasome	2.089263
RNA polymerase	2.019867
Nonhomologous end-joining	1.882127
Circadian rhythm	1.484552
Ribosome	1.321576
Ovarian steroidogenesis	1.002110

## Data Access

Project name: NFP. Project home page: https://cran.r-project.org/web/packages/NFP/. Operating system(s): platform independent. Programming language: R. Other requirements: R 3.2.0 or higher. License: GNU GPLv2. Any restrictions to use by nonacademics: none.
